# An observational study of hospitalized COVID-19 patients with cancer in San Diego county

**DOI:** 10.2217/fon-2021-1116

**Published:** 2022-02-02

**Authors:** David J Hermel, Jason Cham, Samantha R Spierling Bagsic, Lee K Hong, Carrie L Costantini, James R Mason, Alan Saven, Darren S Sigal

**Affiliations:** ^1^Division of Hematology & Oncology, Scripps Clinic, La Jolla, CA 92037, USA

**Keywords:** cancer, COVID-19, hematologic malignancy, lung cancer, lung metastasis, SARS-CoV-2, systemic anti-cancer therapy

## Abstract

**Aim:** To delineate clinical correlates of COVID-19 infection severity in hospitalized patients with malignancy. **Methods:** The authors conducted a retrospective review of all hospitalized patients with a hematologic and/or solid tumor malignancy presenting to the authors' institution between 1 March 2020 and 5 January 2021, with a laboratory confirmed diagnosis of COVID-19. Univariate and multivariate logistic regression analyses were used to determine associations between specific severity outcomes and clinical characteristics. **Results:** Among 2771 hospitalized patients with COVID-19, 246 (8.88%) met inclusion criteria. Patients who were actively receiving treatment had an increased rate of death following admission (odds ratio [OR]: 2.7). After adjusting for significant covariates, the odds ratio increased to 4.4. Patients with cancer involvement of the lungs had a trend toward increased odds of death after adjusting for covariates (OR: 2.3). **Conclusions:** Among COVID-19 positive hospitalized cancer patients, systemic anti-cancer therapy was associated with significantly increased odds of mortality.

## Plain language summary

Though cancer is a biologically heterogenous disease with a wide spectrum of clinical features and behavior, accumulating evidence suggests that cancer patients are at greater susceptibility to COVID-19 infection and more likely to experience morbidity and mortality from COVID-19 infection than non-cancer patients. In this study, the authors reviewed the clinical characteristics of patients with a diagnosis of cancer hospitalized with COVID-19 to assess potential correlates of COVID-19 severity in this population. Notably, analysis of the hospital data revealed a statistically significant increased incidence of mortality in cancer patients who were receiving systemic anti-cancer treatment, including chemotherapy, immunotherapy or targeted therapy, than in those not on therapy. Likewise, there was a trend toward increased mortality in those with either primary or metastatic tumor involvement of the lung compared with those without lung involvement.

The COVID-19 outbreak, facilitated by the rapid spread of the SARS-CoV-2, has been a significant cause of morbidity and mortality since its initial description in December 2019 [1]. Given its virulence and wide spectrum of clinical manifestations, worldwide research efforts have sought to delineate risk factors for severe infection [2]. Specific clinical features associated with poor outcomes include advanced age, male sex and medical comorbidities, including diabetes, coronary artery disease, end stage renal disease on dialysis and active cancer [3–5].

Several studies have highlighted more severe SARS-CoV-2 infection and a higher risk of mortality among patients with cancer [4,5]. In one analysis, patients with cancer were found to have an odds ratio (OR) as high as 4 for in-hospital mortality [4]. Given the inherent predisposition to severe infection in patients with cancer, determining the risk of initiating anti-cancer therapy vis-à-vis increasing susceptibility to a life-threatening virus requires a nuanced understanding of the clinical and treatment-related factors in this population that are associated with overall worse outcomes. Balancing treatment-related immunosuppression, frequent healthcare interaction, tumor-specific characteristics and COVID-19 risks must be carefully considered.

The authors conducted a retrospective observational study of all patients with a diagnosis of cancer hospitalized for COVID-19 within one of five Scripps Health hospitals in San Diego county, with the objective of identifying potential correlates for severe COVID-19 infection, defined as admission to the intensive care unit (ICU), intubation or death.

## Methods

### Study design

The study was approved by the Scripps Health Institutional Review Board and the authors were compliant with all ethical protocols. Patients hospitalized within the regional Scripps Health hospital system in San Diego county from 1 March 2020 to 5 January 2021, with a PCR-confirmed diagnosis of COVID-19 were included in the initial dataset. COVID-19 was confirmed by the testing platforms in use at Scripps Medical Laboratories, including the Hologic and Abbott ID now SARS-CoV-2 PCR assays. The threshold of positivity was standardized across Scripps laboratories at different hospitals using the same universal US FDA–approved testing platforms with concordant testing treatment algorithm. Only patients who were hospitalized with a positive COVID-19 test during their hospital admission were included in the initial analysis.

Individuals meeting these inclusion criteria with multiple hospitalizations were included only for the initial hospitalization following a positive COVID test. Patients with do-not-resuscitate orders were included in this analysis. Relevant laboratory and clinical data were extracted from the Scripps Health electronic medical record. A cancer diagnosis was determined based on review of the International Classification of Diseases, Tenth Revision, codes (ICD-10 codes) listed within each patient's medical record during the hospitalization of interest. Patients with non-melanoma skin cancers and *in situ* disease were excluded from analysis.

Clinical severity outcomes of interest included admission to the ICU, intubation during hospitalization, readmission and death. Death was defined as any mortality within the study period, including post-hospitalization. Systemic anti-cancer therapy included cytotoxic chemotherapy, immunomodulators, immune checkpoint inhibitors and other targeted therapies. In this study, androgen deprivation therapy, anti-estrogen therapy and steroids were not included as anti-cancer therapy, as compliance and use of these medications were not verifiable in the medical record. Somatostatin analogs and abiraterone acetate were included. Extracted clinical information included demographic details, baseline clinical comorbidities, laboratory results and medications administered within a patient's hospitalization. Demographic details included age, BMI, sex and ethnicity (grouped as either Hispanic or non-Hispanic).

### Statistical analysis

Demographic and clinical characteristics were descriptively summarized for all patients meeting the inclusion criteria. Categorical data were described as frequencies and percentages, and continuous data were described as means and standard deviations if normally distributed and medians and interquartile ranges otherwise. Associations between each outcome of interest and tumor type, hematologic versus solid malignancy, metastatic involvement (with or without lung involvement) and use of active systemic anti-cancer therapy (treatment within 3 months of or during admission) were determined using univariable logistic regression analyses and odds ratios, and 95% confidence intervals were reported. Demographic and clinical covariates were identified as any such variable that significantly predicted each of the outcome variables in univariable logistic regression analyses, and all significant predictors were then included in multivariable analyses for each outcome and backwards reduced to only significant characteristics. These significant characteristics were then included in multivariable models as fixed effects for each adjusted outcome analysis, and adjusted odds ratios and confidence intervals were reported. All p-values reported are two-tailed, and p < 0.05 was considered statistically significant. All analyses were conducted using R v. 4.0.3 and figures were generated using GraphPad Prism v. 9.

## Results

Among a total of 2771 hospitalized patients, 246 (8.88%) met the inclusion criteria. The average age was 72.95 (13.89) years, 50.4% were male, 35.4% were Hispanic and the average BMI was 27.85 (7.03). The majority of patients (84.9%) had solid tumors, with the most prevalent being prostate carcinoma (19.1%) and breast carcinoma (17.9%). Overall, 48 (19.5%) were admitted to the ICU, 29 (11.8%) were intubated and 62 (25.2%) died during or after their hospital admission. In total, 83 (33.7%) patients were re-admitted. The majority of patients had localized disease, with 19.9% having metastatic disease and 13.4% having primary or metastatic lung involvement. In total, 17.1% were receiving systemic anti-cancer treatment. Table 1 contains complete cohort demographic and malignancy characteristics.

**Table 1.** Patient demographics and cancer characteristics.

Demographic (n = 246)	n (%)
Sex: male	124 (50.4%)
Ethnicity: Hispanic	87 (35.4%)
	**Mean (SD)**
Age at admission	73.0 (13.9)
BMI at admission (# NA: 14)	27.9 (7.0)

Cancer characteristic	n (%)
Solid	209 (85%)
Metastatic	49 (19.9%)
Lung involvement	33 (13.4%)
Treatment	42 (17.1%)
– Cytotoxic	23 (9.3%)
– Immune checkpoint	2 (0.8%)
– Other	17 (6.9%)

NA: Not available.

Among patients receiving systemic anti-cancer therapy, 54.8% received cytotoxic chemotherapy, 4.8% received immune checkpoint inhibitors, and 40.5% received other agents (including lanreotide, ibrutinib, revlimid, palbociclib, rituximab, abiraterone, sunitinib, dabrafenib, trametinib, ixazomib, dasatinib, trastuzumab, bevacizumab, blinatumomab and venetoclax).

On univariable analysis, patients who were receiving systemic anti-cancer treatment had an increased rate of death (42.9 vs 21.6%; OR: 2.7 [1.3–5.5]; p = 0.005). Moreover, there was a trend toward increased mortality in those with lung involvement of their tumor (36.4 vs 21.6%; OR: 2.1 [0.9–4.5]; p = 0.072). Other outcome variables, including ICU stay, intubation and re-admission, were not associated with systemic anti-cancer therapy, metastatic disease or tumor type (solid vs hematologic) in univariable analyses. Please see Table 2 for patient outcomes stratified by cancer characteristics.

**Table 2.** Patient outcomes by cancer characteristics.

Solid vs Liquid	Liquid (n = 37)	Solid (n = 209)	Unadjusted		Adjusted^†^	
Outcome	n (%)	n (%)	OR (95% CI)	p-value	OR (95% CI)	p-value
ICU	8 (21.6%)	40 (19.1%)	0.86 (0.38–2.14)	0.726	0.98 (0.43–2.46)	0.958
Intubated	5 (13.5%)	24 (11.5%)	0.83 (0.32–2.6)	0.724	1.22 (0.44–4.02)	0.721
Death	12 (32.4%)	50 (23.9%)	0.66 (0.31–1.44)	0.274	0.54 (0.23–1.3)	0.157
Death during hospitalization	12 (32.4%)	37 (17.7%)	0.45 (0.21–1)	0.042	0.51 (0.21–1.27)	0.134
Re-admission	12 (32.4%)	71 (34%)	1.07 (0.52–2.33)	0.855	1.1 (0.52–2.43)	0.814

Among solid tumors: metastatic vs not:	Not metastatic (n = 160)	Metastatic (n = 49)	Unadjusted		Adjusted^†^	
Outcome	n (%)	n (%)	OR (95% CI)	p-value	OR (95% CI)	p-value
ICU	32 (20%)	8 (16.3%)	0.78 (0.31–1.76)	0.568	0.69 (0.27–1.57)	0.395
Intubated	19 (11.9%)	5 (10.2%)	0.84 (0.27–2.24)	0.748	0.93 (0.28–2.67)	0.892
Death	35 (21.9%)	15 (30.6%)	1.58 (0.76–3.19)	0.212	1.92 (0.83–4.38)	0.1204
Re-admission	56 (35%)	15 (30.6%)	0.82 (0.4–1.61)	0.571	0.78 (0.37–1.57)	0.498

Lung involvement vs not:	No lung involvement (n = 176)	Lung involvement (n = 33)	Unadjusted		Adjusted^†^	
Outcome	n (%)	n (%)	OR (95% CI)	p-value	OR (95% CI)	p-value
ICU	31 (17.6%)	9 (27.3%)	1.75 (0.71–4.04)	0.200	1.74 (0.7–4.03)	0.212
Intubated	19 (10.8%)	5 (15.2%)	1.48 (0.46–4.03)	0.474	2.01 (0.59–6.02)	0.229
Death	38 (21.6%)	12 (36.4%)	2.08 (0.92–4.55)	0.072	2.28 (0.91–5.63)	0.074
Re-admission	59 (33.5%)	12 (36.4%)	1.13 (0.51–2.43)	0.752	0.88 (0.37–1.96)	0.751

Receiving treatment vs not:	Not receiving treatment (n = 204)	Receiving treatment (n = 42)	Unadjusted		Adjusted^†^	
Outcome	n (%)	n (%)	OR (95% CI)	p-value	OR (95% CI)	p-value
ICU	39 (19.1%)	9 (21.4%)	1.15 (0.49–2.52)	0.731	0.95 (0.39–2.13)	0.908
Intubated	24 (11.8%)	5 (11.9%)	1.01 (0.32–2.64)	0.980	0.76 (0.23–2.08)	0.613
Death	44 (21.6%)	18 (42.9%)	2.73 (1.35–5.47)	0.005	4.45 (1.9–10.65)	0.001
Re-admission	68 (33.3%)	15 (35.7%)	1.11 (0.54–2.2)	0.766	0.95 (0.45–1.94)	0.887

Among those receiving treatment: cytotoxic vs other	Other (n = 19)	Cytotoxic (n = 23)	Unadjusted		Adjusted^†^	
Outcome	n (%)	n (%)	OR (95% CI)	p-value	OR (95% CI)	p-value
ICU	6 (31.6%)	3 (13%)	0.33 (0.06–1.46)	0.156	0.3 (0.06–1.39)	0.138
Intubated	3 (15.8%)	2 (8.7%)	0.51 (0.06–3.41)	0.486	0.43 (0.05–3.07)	0.404
Death	9 (47.4%)	9 (39.1%)	0.71 (0.21–2.45)	0.592	0.96 (0.22–4.2)	0.952
Re-admission	6 (31.6%)	9 (39.1%)	1.39 (0.39–5.2)	0.612	1.35 (0.37–5.12)	0.647

† See Table 3 listing covariates included in the adjusted model for each outcome.

ICU: Intensive care unit; OR: Odds ratio.

**Figure 1.**
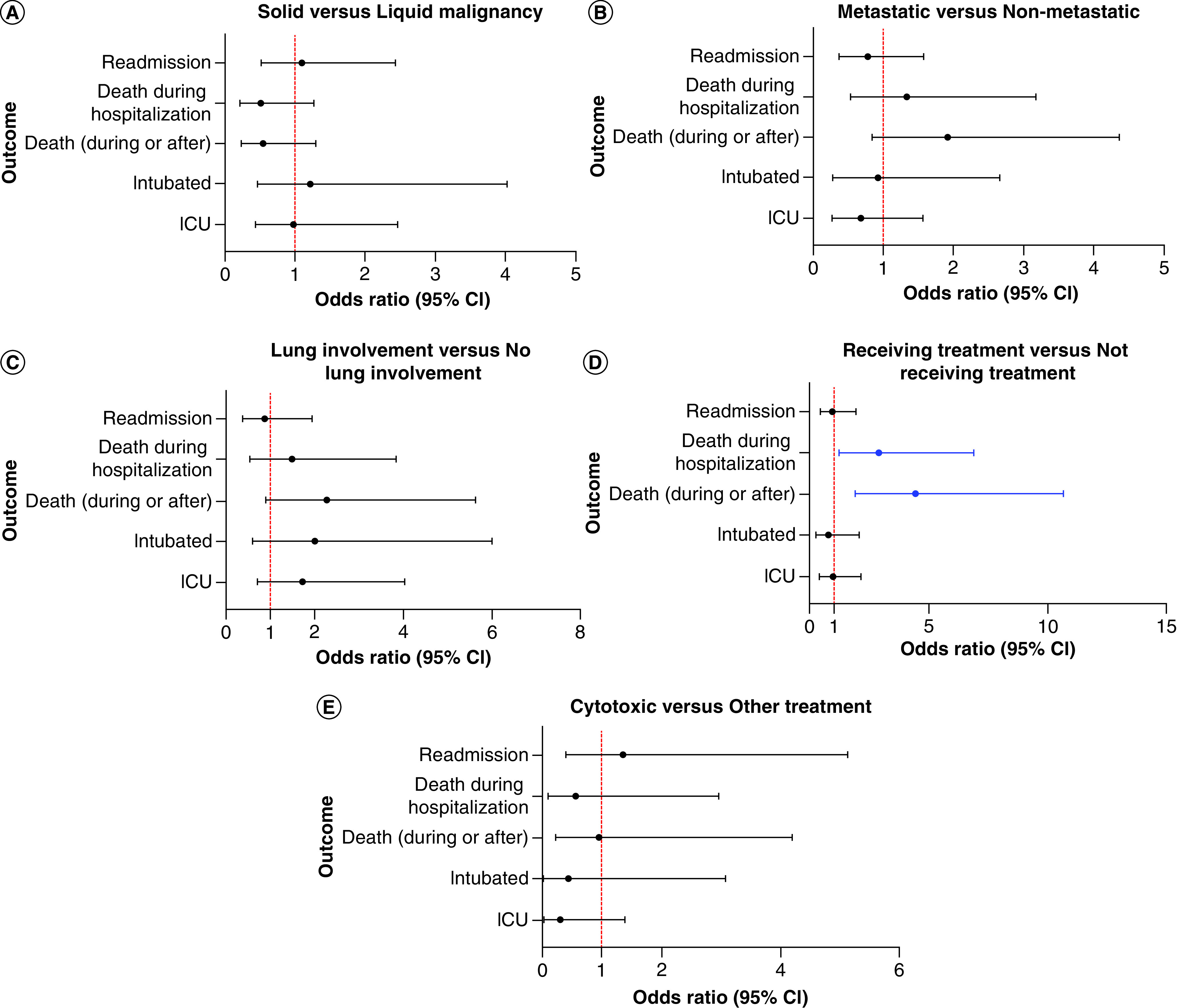
Covariate adjusted odds ratios. **(A)** Solid versus liquid malignancy, **(B)** metastatic versus non-metastatic, **(C)** lung involvement versus no lung involvement, **(D)** receiving treatment versus not receiving treatment and **(E)** cytotoxic versus other treatment. ICU: Intensive care unit.

These findings on univariate analysis persisted when adjusting for significant covariates (see Table 3 for covariates included for each outcome). Those receiving active systemic cancer treatment had increased odds of death relative to those not receiving treatment (OR: 4.4 [1.9–10.7]; p = 0.001) after adjusting for significant covariates including but not limited to sex, age at admission and comorbidities of atrial fibrillation, deep vein thrombosis (DVT) or thrombocytopenia. Lung involvement also trended toward increased odds of death adjusting for these covariates (OR: 2.3 [0.9–5.6]; p = 0.07). No other statistically significant associations were observed in covariate adjusted analyses. Please see Figure 1 for plots that depict the covariate adjusted ORs for various outcomes.

**Table 3.** Significant covariates for inclusion in adjusted models for each outcome.

Outcome	Characteristic	Odds ratio (OR) (95% CI)	p-value
Intensive care unit	Anemia	1.98 (1.02–4.03)	0.049
Intubated	Hispanic ethnicity	3.22 (1.42–7.6)	0.006
	Asthma	4.57 (1.26–15.19)	0.015
	Anemia	2.87 (1.16–8.17)	0.031
Death	Sex	2.22 (1.18–4.27)	0.015
	Age at admission	1.03 (1–1.06)	0.032
	Atrial fibrillation	2.16 (1.08–4.29)	0.029
	Deep vein thrombosis	3.45 (1.38–8.7)	0.008
	Thrombocytopenia	2.74 (1.39–5.42)	0.004
Re-admission	Sex	0.48 (0.28–0.83)	0.009
	Chronic obstructive pulmonary disease or emphysema	2.37 (1.23–4.57)	0.010

## Discussion

In this single-institution, multi-hospital, retrospective, observational study of hospitalized COVID-19 cancer patients in San Diego county, there was a significant association between systemic anti-cancer therapy and death, even after adjusting for significant covariates. Moreover, there was a trend toward increased mortality in patients with either primary or metastatic tumor involvement of the lung.

This descriptive study helps better characterize the heterogenous population with neoplastic diseases hospitalized with COVID-19 and highlights specific subgroups of patients with potentially worse outcomes. Those receiving systemic anti-cancer therapy, which includes a broad range of cytotoxic, targeted and immunomodulatory drugs, were more likely to die during the study period. One potential rationale for this correlation is the treatment-related effects on immunity that may dampen host response to SARS-CoV-2. Alternatively, acquiring COVID-19 could have resulted in dose delays and treatment discontinuation that enabled tumor progression and death. Another consideration is that patients who require anti-cancer treatment likely have more advanced disease and are more susceptible to death. In this study, no independent analysis of cancer progression-related deaths and deaths from other causes was conducted. Thus, although the results of this analysis suggest an association between death and recent initiation of systemic anti-cancer therapy in hospitalized COVID-19 patients, they do not prove causality.

Initial nationwide data analysis from China at the start of the pandemic showed a higher risk of COVID-19 in cancer patients than in non-cancer patients (1 vs 0.29% per 100,000 people) as well as poorer outcomes in this population when compared with non-cancer patients (39 vs 8%) [6]. One rationale for poorer outcomes is the use of systemic therapy, which can dampen host response to infection [7]. A study by Sng *et al.* showed that patients undergoing cancer therapy within 60 days of a diagnosis of COVID-19 had a hazard ratio of death of 2.3 [8]. Other studies did not find evidence that cancer patients on cytotoxic chemotherapy or other anti-cancer treatments had an increased risk of mortality from COVID-19 [9,10]. Comparisons between different patient populations is difficult and requires specific attention to the treatment regimens included in the analysis, the time studied during the pandemic [11], the vaccination status of patients and the proportion of hematologic versus solid tumor malignancy.

Despite the genomic and phenotypic heterogeneity of cancer and the variations in clinical course among cancer types, accumulating evidence suggests that a diagnosis of cancer is an independent risk factor for COVID-19-related morbidity and mortality. Depending on the study, cancer has been associated with estimated COVID-19 mortality rates ranging from 13 to 40.5% [3,12–16]. Moreover, when stratified among tumor types and characteristics, data suggest worse outcomes in patients with hematologic malignancy or lung cancer [14–16]. Consistent with other studies, our data do support a trend toward worse outcomes in patients who have lung involvement of their tumor, as these patients are more likely to have respiratory manifestations following a COVID-19 diagnosis [17].

In summary, this study identifies a subgroup of hospitalized COVID-19 positive cancer patients – those receiving systemic anti-cancer therapy – that appear to be at risk of increased mortality. Though the study does not prove causality, it does help frame a discussion regarding the risks and benefits of initiating cancer-directed treatment during a global pandemic. Further validation of these findings in a larger sample could impact therapeutic decision making during the COVID-19 pandemic and help optimally stratify those at increased risk of COVID-19-related complications.

**Summary points**

- Systemic anti-cancer therapy was associated with significantly increased odds of mortality in patients hospitalized with COVID-19 infection.
- Patients hospitalized for coronavirus disease 2019 with malignancy in the lung had a trend toward increased mortality.
